# MethylPCA: a toolkit to control for confounders in methylome-wide association studies

**DOI:** 10.1186/1471-2105-14-74

**Published:** 2013-03-02

**Authors:** Wenan Chen, Guimin Gao, Srilaxmi Nerella, Christina M Hultman, Patrik KE Magnusson, Patrick F Sullivan, Karolina A Aberg, Edwin JCG van den Oord

**Affiliations:** 1 Department of Biostatistics, School of Medicine, Virginia Commonwealth University, Richmond, VA, USA; 2 Center for Biomarker Research and Personalized Medicine, School of Pharmacy, Virginia Commonwealth University, Richmond, VA, USA; 3 Swedish Schizophrenia Consortium, Department of Medical Epidemiology and Biostatistics, Karolinska Institutet, Stockholm, Sweden; 4 Department of Genetics, University of North Carolina at Chapel Hill, Chapel Hill, NC, USA

**Keywords:** Principal component analysis, Methylome-wide association studies, Eigen-decomposition, Association test, MBD-seq

## Abstract

**Background:**

In methylome-wide association studies (MWAS) there are many possible differences between cases and controls (e.g. related to life style, diet, and medication use) that may affect the methylome and produce false positive findings. An effective approach to control for these confounders is to first capture the major sources of variation in the methylation data and then regress out these components in the association analyses. This approach is, however, computationally very challenging due to the extremely large number of methylation sites in the human genome.

**Result:**

We introduce MethylPCA that is specifically designed to control for potential confounders in studies where the number of methylation sites is extremely large. MethylPCA offers a complete and flexible data analysis including 1) an adaptive method that performs data reduction prior to PCA by empirically combining methylation data of neighboring sites, 2) an efficient algorithm that performs a principal component analysis (PCA) on the ultra high-dimensional data matrix, and 3) association tests. To accomplish this MethylPCA allows for parallel execution of tasks, uses C++ for CPU and I/O intensive calculations, and stores intermediate results to avoid computing the same statistics multiple times or keeping results in memory. Through simulations and an analysis of a real whole methylome MBD-seq study of 1,500 subjects we show that MethylPCA effectively controls for potential confounders.

**Conclusions:**

MethylPCA provides users a convenient tool to perform MWAS. The software effectively handles the challenge in memory and speed to perform tasks that would be impossible to accomplish using existing software when millions of sites are interrogated with the sample sizes required for MWAS.

## Background

Methylation studies are a promising complement to genetic studies of DNA sequence variation. First, as methylation is typically associated with transcriptional repression [1,2], it may account for additional variation in disease susceptibility. Second, methylation studies can shed a unique light on clinical phenomena [3] such as sex differences [4,5], genotype environment interactions [6], and disease course over time [7]. Finally, methylation sites are potential new drug targets [8] and have good properties from a translational perspective such as being stable and enabling cost-effective assays in biosamples that are relatively easy to collect [9].

Because detailed prior biological knowledge is lacking, it will be critical to perform methylome-wide association studies (MWAS) to detect disease relevant sites [10,11]. The most comprehensive approach uses next-generation sequencing (NGS) to interrogate DNA methylation on a genome-wide basis after bisulfite conversion of unmethylated cytosines. The single base resolution afforded by bisulfite sequencing is attractive but currently this approach is not economically feasible for the large sample sizes required for MWAS [12]. In a cost-effective alternative the genome is fragmented and the methylated fragments are bound to antibodies [13] (e.g. MeDIP) or other proteins [14] (e.g. MBD-seq) with high affinity for methylated DNA. The unmethylated genomic fraction is washed away, and then only the methylation-enriched portion is sequenced. A final option involves arrays. Examples are the commercially available Infinium system from Illumina [15] that interrogates >450,000 loci or genome-wide tiling arrays and the 45 million probe array set from Affymetrix [16] that offers more comprehensive coverage of the methylome.

In addition to technical factors associated with processing samples, in MWAS there are many possible differences between cases and controls that may affect the methylome. Examples include differences in life style, diet, and medication use. As these confounding variables correlate with both the dependent (case–control status) and independent variable (methylation status) they will cause spurious associations that are not of direct substantive interest because they are unrelated to disease processes. Controlling for confounders in MWAS is critical to avoid a flood of false positive findings. If measured, such variables can be regressed out. However, the list of potential confounders is long, only a subset of these variables will have been measured, and many confounders may simply be unknown. Statistical methods that first capture the major sources of variation in the methylation data, and then regress out these components when performing the association analyses may provide an effective solution. However, because of the ultra high dimension of methylation data (e.g. the methylation of DNA cytosine residues at the carbon 5 position (5^me^C) occurs in the vast majority of cases at CpG sites of which already 27 million exist in the human reference genome), standard statistical packages or existing software for the analysis of large scale methylation data cannot be used [17-20]. To address the computational challenges we developed a toolkit called MethylPCA that is specifically designed to control for confounders in MWAS.

MethylPCA uses principal component analysis (PCA) to capture the major sources of variation in the methylation data. Although other options exist, PCA has the advantage of being well developed (e.g. algorithms exist that enable PCA on ultra-high dimensional data), is computationally efficient, and has already been successfully applied to high-dimensional biological data [21]. EIGENSOFT [21,22] also performs PCA. However, 1) MethylPCA provides an adaptive procedure designed to combine methylation data of neighboring sites into larger blocks prior to PCA; 2) Even after this data reduction step, calculation of the input matrix for the PCA would be prohibitive in terms of memory and CPU time for large sample sizes. MethylPCA allows partitioning the data into a user-specified number of sets to compute sub-matrices in parallel on a cluster and then assemble those to obtain the complete input matrix; 3) To enable a complete and flexible data analysis pipeline, MethylPCA provides options to perform PCA based on the covariance matrix and/or correlation matrix and includes an association testing procedure where covariates such as the calculated principal component scores (PCs) can be regressed out; 4) EIGENSOFT is designed to process categorical SNP data while our software can work on the quantitative methylation data.

## Implementation

The design philosophy of MethylPCA is to build small independent executable components first and then combine them to perform complex tasks. There are two advantages for this design style: 1) Easy to debug. Each component can be debugged independently. 2) Flexible to use. Each component can be used either independently or combined together. MethylPCA consists of three major components that can be run individually or as a pipeline:

1) Creating blocks. This procedure adaptively combines inter-correlated methylation data from adjacent sites.

2) PCA. It performs PCA on the methylation data and outputs the calculated PC scores, eigenvalues and loadings.

3) Association test. It performs association tests using multiple linear regression with optional supplied covariates (e.g., age, gender) and the PC scores calculated from the PCA procedure. It outputs the test statistics and p-values, as well as a QQ plot.

A user-friendly interface is provided in the form of a parameter file that controls which and how procedures are performed (see Additional file 1 for detailed description of software). For example, the above three procedures can be performed sequentially or individually by putting the parameters corresponding to the procedures in the parameter file. Each procedure has multiple parameters to be set in the parameter file in order to run it properly. The computational and I/O intensive parts of MethylPCA are implemented in C++ and the remainder in the R language.

### Creating blocks

In MWAS correlations often exist between adjacent sites. Rather than using a sliding window of arbitrary length, MethylPCA uses an adaptive algorithm that combines methylation data based on the observed inter-correlations. A benefit of creating “blocks” is that the data reduction speeds up subsequent analyses, e.g. the PCA procedure. The use of blocks may also prevent that the results of PCA are dominated by a limited number of regions containing highly correlated sites as well as improve the signal to noise ratio because a sum of substantially inter-correlated measurements is known to be more reliable than the individual measurements separately [23]. Because there may be regions in a chromosome where there is differential methylation in just one CpG site, sites that are uncorrelated with neighboring sites are also kept by forming “blocks” that consist of a single CpG sites only.

Correlations between sites can occur for different reasons. For example in MBD-seq neighboring CpGs will be highly correlated because they are largely covered by the same DNA fragments. Correlations can also occur because of biological phenomenon [24]. To account for these different causes, MethylPCA allows creating blocks in two stages. The first stage combines the sites that are largely covered by the same fragments to form the level 1 block data. Next the level 1 block data is combined to capture the “biological” correlations to form the level 2 block data.

Sometimes excluding some sites in the analysis is useful, e.g. those sites with low coverage or that are in repeats. There is an option to provide files that specify which sites are included or excluded. The computing time for creating blocks is approximately proportional to *n × p*, where *n* is the number of subjects and *p* is the number of sites. Because a block merged from multiple sites is processed as a single unit in the following analysis, the word “site” in the following text may either refer to a single CpG site, or a block containing multiple neighboring CpG sites.

Three parameters control the block creation. The first is a threshold for the average correlations inside a block denoted by *t*_*1*_. The second is a threshold denoted by *n*_*t*_ for the number of new sites added to the block that have a mean correlation with sites already in the block below a third threshold labeled *t*_*2*_*.* The merging process of a block stops if 1) the average correlations in the block is below *t*_*1*_ or *n*_*t*_ new sites are merged having correlations with sites already in the block below *t*_*2*_. The output block data uses the mean of all methylation values inside the block to represent each block and stores the related block information such as the beginning of the block, the end of the block and the average correlation within the block in a separate file.

### Principal component analysis (PCA) when *p* >>*n*

PCA is typically performed on the *p × p* sample covariance matrix $C=\frac{1}{n-1}X^{T}X$, where *X* is the *n* × *p* data matrix, *n* the number of subjects and *p* the number of methylation sites. When *p* is much larger than *n*, direct eigen-decomposition of *C* is no longer computationally feasible. However, we can obtain the same PCA results through eigen-decomposition of the much smaller *n × n* matrix $M=\frac{1}{n-1}XX^{T}$, sometimes called principal coordinate analysis [25].

We assume that *X* has been centered by subtracting the mean of each column from the original observations. The sample covariance matrix $C=\frac{1}{n-1}X^{T}X$ is a *p × p* matrix. Suppose *λ*_*1*_, *λ*_*2*_, …, *λ*_*r*_ are the positive eigenvalues in descending order and *v*_*1*_, *v*_*2*_, … *v*_*r*_ are the corresponding orthonormal eigenvectors of *C*, with subscript *r* being the rank of *X*. Then *PC*_*i*_ *= Xv*_*i*_ is the *n*-dimensional column vector of the *i*th principle component (PC) scores of methylation data across *p* sites for all *n* subjects, where the elements in *v*_*i*_ are also called the loadings of *PC*_*i*_. When *p* >>*n* it can be computationally infeasible to conduct the PCA through eigen-decomposition of matrix C. Instead, we can calculate PC_i_ by using the *n* × *n* matrix $M=\frac{1}{n-1}XX^{T}$, which is a similarity matrix or inner product matrix between all subjects. Suppose *α*_*1*_, *α*_*2*_, …, *α*_*r*_ are the positive eigenvalues in descending order and *u*_*1*_, *u*_*2*_, …*u*_*r*_ are the corresponding orthonormal eigenvectors of *M*. Let *U* = [*u*_*1*_, *u*_*2*_, …*u*_*r*_] and *V* = [*v*_*1*_, *v*_*2*_, … *v*_*r*_]. If *U* and *V* are properly chosen when they are not unique, we have (see[25])

(1) $$\lambda_{i}=\alpha_{i}$$

(2) $$PC_{i}=X\;v_{i}=u_{i}\sqrt{\left(n-1\right)\alpha_{i}},i=1,\ldots ,r.$$

So the PC scores can be calculated using *u*_*i*_ and *α*_*i*_. Similarly we have

(3) $$\begin{array}{l} v_{i}=\frac{1}{\sqrt{\left(n-1\right)\alpha_{i}}}{\;X}^{T}u_{i}=\frac{1}{\left(n-1\right)\alpha_{i}}{\;X}^{T}PC_{i},i\\ \;=1,\ldots ,r, \end{array}$$

Therefore, the loadings *v*_*i*_ can also be calculated from *u*_*i*_ and *α*_*i*_. EIGENSOFT [21] employs a similar method to calculate principle components.

In MethylPCA, we compute and store the *n* × *n* matrix *X*, and the PCs are then calculated from the eigenvectors of $M=\frac{1}{n-1}XX^{T}$ (see Equation 2). The loadings are calculated based on the original data matrix *X* and the PCs or the eigenvectors of *M* (see Equation 3). The main computing challenge (both in memory and time) is the calculation of the matrix *XX*^*T*^ that becomes prohibitive for large samples using existing software. To handle this challenge, MethlPCA can calculate user-specified chunks of the matrix *XX*^*T*^ after which the full matrix *XX*^*T*^ is assembled. Because each computing job only works on a specified number of samples loaded into the memory for calculation instead of loading the entire methylation data, this solves the problem of processing large data sets with limited memory. If a cluster is available, each computing job can be executed in parallel to speed up the process. Statistics that are used repeatedly (e.g. means of all sites in the entire sample) are calculated only once and stored to further increase efficiency. PCA based on the correlation matrix is sometimes preferred because PCA on a covariance matrix can be dominated by variables with large variances [26]. MethylPCA provides options to perform PCA based on the correlation matrix or covariance matrix. Even though it is possible to calculate the loadings for each PC, usually we are only interested in the loadings corresponding to the top PC scores. To reduce the computing time, users can specify the number of top principal components for which loadings will be calculated. The computing time of PCs is proportional to *n*^*2*^ *× p* plus the time reading the data into the memory. The computing time for one loading is approximately proportional to *n × p*.

Because covariates that have been measured can be regressed out directly in the MWAS, the motivation for using PCA is typically to control for the unmeasured confounders. To better capture unmeasured confounders and include those together with the measured covariates in the MWAS, it is possible to regress out measured covariates prior to performing the PCA. This could include, for example, technical factors associated with processing samples such as the quantity of genomic DNA starting material or sample batches. This option is implemented using the multiple regression functions from GNU Scientific Library (GSL) [27]. The adjusted methylation data used in the PCA are the residuals after regressing out the measured covariates.

### Association test

To enable a complete data analysis pipeline, we also added the possibility to perform MWAS in MethylPCA through multiple linear regression analysis using functions in GSL [27]. It tests the association between the phenotype and each methylation site while adjusting for covariates. Users can choose which covariates will be included in the association tests, such as age, gender and PCs. The test statistic and the p-values for each site are calculated and stored. Once all test statistics are generated, the genomic control inflation factor lambda is calculated, which is defined as the observed median test statistic value divided by the expected median of a chi-square distribution with 1 degree of freedom [28]. Under the null hypothesis that there is no effect for any site, lambda is close to 1. Finally a QQ (quantile-quantile) plot is produced based on the p-values and the calculated lambda is also displayed. The association tests for different chromosomes can be computed in parallel to decrease CPU time.

### Support for both a single computer and a cluster

An option is provided in the parameter file that controls whether to submit the computing jobs to a cluster or run it sequentially on a single computer. After analyzing the parameter file, all computing jobs will be arranged. Each computing job is written as a line of an executable command with corresponding parameters and is stored into batch files. For example, the block creating procedure can be performed per chromosome, with each command line processing one chromosome in the corresponding batch file.

## Results

### Simulation study

In this simulation study, we illustrate the effectiveness of PCA in correcting confounding factors in the association test. We simulate two types of confounding factors: continuous and dichotomous. We assume that the same number of cases and controls are collected in the case–control data and let *y*_*i*_ denote the disease status of the *i*th subject, 1 for case and 0 for control, *i* = 1, …, *n*. We simulate *K* confounding factors. The *k*th confounding factor for subject *i* as a continuous variable is simulated as follows:

$$F_{\mathrm{ik}}=b_{k}\times y_{i}+m_{\mathrm{ik}},i=1,\ldots ,n,1\leq k\leq K,$$

where *m*_*ik*_ follows a normal distribution with mean 0 and variance *σ*^*2*^, *b*_*k*_ is a parameter which together with *σ*^*2*^ controls the correlation between the *k*th factor and the case–control status *y*_*i*_ (see Additional file 2). If the *k*th confounding factor is dichotomous, such as smoking/non-smoking status, the distribution of the confounding factor is simulated as:

$$\begin{array}{l} \mathrm{Pr}\left(F_{\mathrm{ik}}=1|y_{i}=1\right)=p_{1},\mathrm{Pr}\left(F_{\mathrm{ik}}=1|y_{i}=0\right)=p_{2,}\\ \;i=1,\ldots ,n,1\leq k\leq K, \end{array}$$

where *p*_*1*_ and *p*_*2*_ control the correlation between the *k*th factor and the case–control status *y*_*i*_ (see Additional file 2). The inclusion of the case–control status in the above models makes sure that there are correlations between the outcome and the confounding factors.

Next, we introduce correlations between the confounding factor and the methylation levels. First we sample the number of CpG sites in each of the 22 chromosomes from a Poisson distribution with the mean of 4,000. Let *x*_*ij*_ be the methylation level of the *i*th subject on the *j*th CpG site, then it is simulated using the following formula:

$$\begin{array}{l} x_{\mathrm{ij}}=a_{j}+\sum_{k=1}^{K}F_{\mathrm{ik}}l_{\mathrm{kj}}+e_{\mathrm{ij}},i=1,\ldots ,n,j=1,\ldots ,J,\\ \;k=1,\ldots ,K \end{array}$$

where *a*_*j*_ is the base level for site *j*. *e*_*ij*_ is the normally distributed error with mean 0 and variance *δ*^*2*^, *J* is the total number of CpG sites. *l*_*kj*_ is 0 or 1 defining the influence range of each confounding factor. If *l*_*kj*_ = 1, then the *k*th confounding factor has effects on site *j*, otherwise there is no effect. We set the influence range of the *k*th confounding factor to cover all sites of three chromosomes, from chromosome (*k*-1) × 2 + 1 to chromosome (*k*-1) × 2 + 3. This means that each factor influences 3 chromosomes and there is one overlapped chromosome influenced by two factors. To ensure non-negative methylation levels, all levels are subtracted by the smallest value in the data matrix *X*.

500 cases and 500 controls were simulated in each data set with 5 confounding factors. We did six simulations in which different combinations of continuous and dichotomous confounding factors were used (see Table 1). We set *b*_*k*_ = 4 and *σ* = 10 for continuous factors so that the correlation between the continuous confounding factor and the case–control status was about 0.2. We set *p*_*1*_ = 0.6 and *p*_*2*_ = 0.4 so that the correlation between the dichotomous confounding factor and the case–control status was also 0.2. *a*_*j*_ were uniformly sampled from 0 to 100. *δ* was set to 40. We applied MethylPCA on the simulated data sets and extracted the top PCs after examining the Scree plot, i.e., plot of eigenvalues. For comparison, we performed association tests with and without the top PCs.

**Table 1:** The comparison of the genomic control inflation factor of association tests with and without top PCs

**Association test**	0c + 5d^*^	**1c + 4d**	**2c + 3d**	**3c + 2d**	**4c + 1d**	**5c + 0d**
without PCs^†^	2.962	3.199	2.979	2.987	2.873	2.949
with PCs^‡^	1.006	1.008	1.009	1.004	1.009	0.996

^*^The number of continuous confounding factors (followed with c) and the number of dichotomous confounding factors (followed with d) in the simulated data set. ^†^The association test without incorporating the top PCs as covariates. ^‡^The association test incorporating the top PCs as covariates.

**Figure 1:**
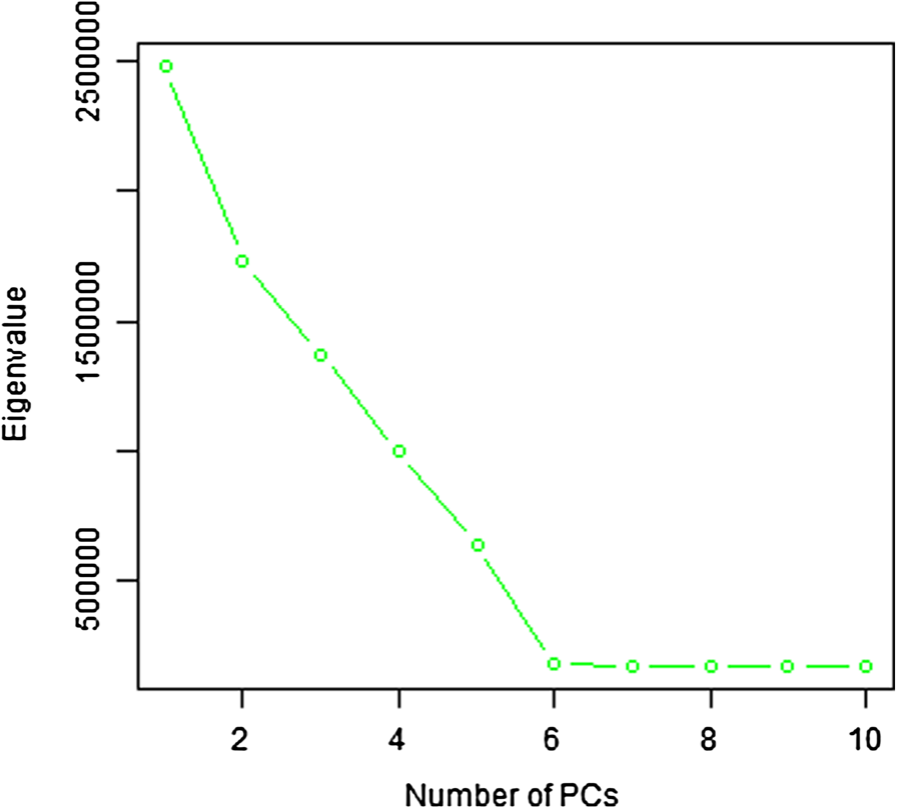
Scree test on a simulated data set with the top 10 eigenvalues.

**Figure 2:**
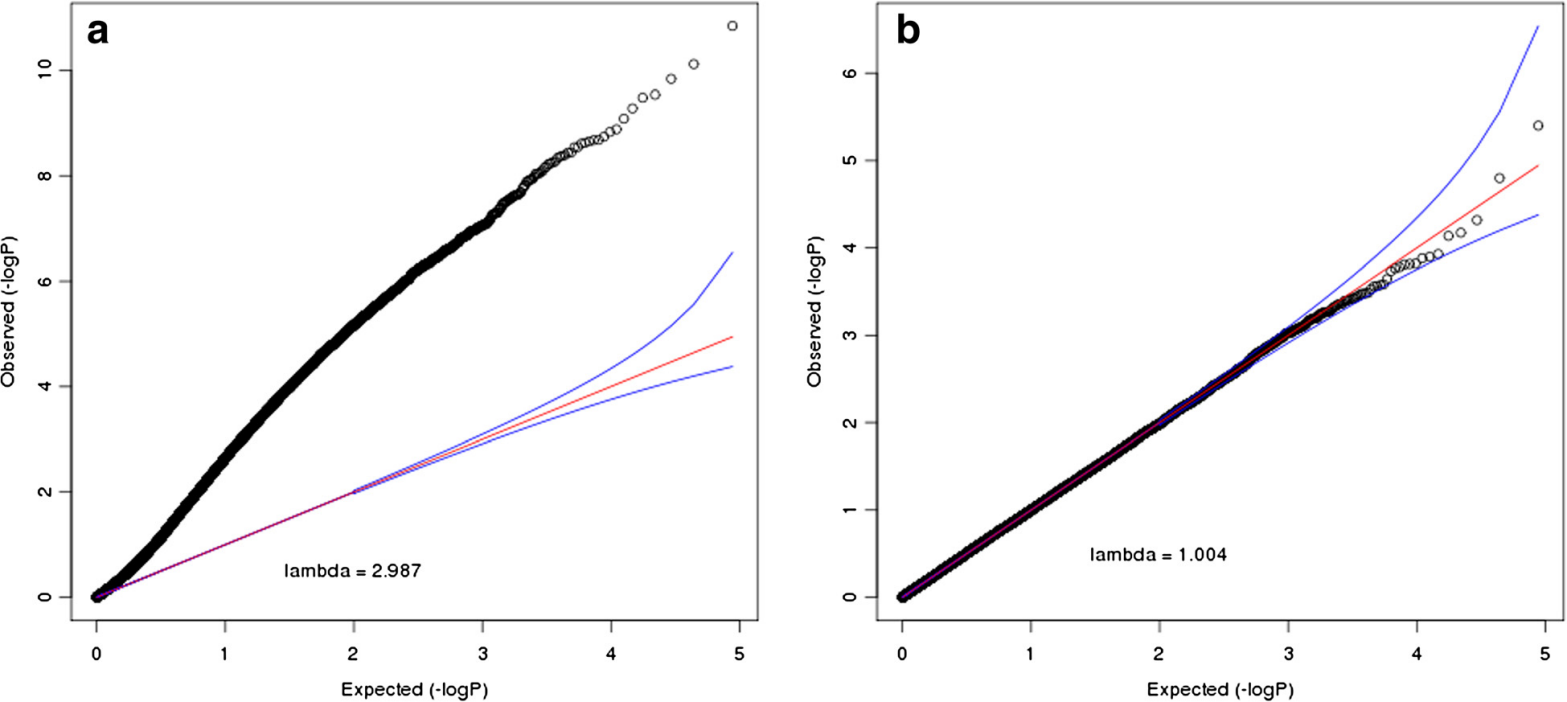
**QQ plot and the lambda values of the association tests before PCA and after PCA on a simulated data set.** Figure 2 (**a**) shows the QQ plot and lambda without including PCs in the association test. Figure 2 (**b**) shows the QQ plot and lambda including PCs in the association test.

Figure 1 shows the Scree plot of the top 10 eigenvalues of the data set with 3 continuous confounding factors and 2 dichotomous confounding factors. This plot clearly captures the number of factors. Therefore the top 5 PCs are included in the following association test. Figure 2 shows the QQ plot of association tests of the same data set with and without incorporating the top PCs. It can be seen that after applying PCs into the testing model, the distribution of p-values is consistent with the null model. Results of other simulations have the similar pattern. Table 1 shows the comparison of lambda values under different simulation settings. We can see that, after regressing out PCs, the lambda is restored to be very close to 1. This indicated that MethylPCA controlled for confounders well.

### MBD-seq MWAS in 1,500 samples

This study includes 750 schizophrenia cases and 750 controls, as well as 75 technical duplicates. For a detailed description of this study and the data analysis pipeline see [29]. In summary, this study is part of a large ongoing project entitled “*A Large-Scale Schizophrenia Association Study in Sweden*”. The project is supported by grants from NIMH and the Stanley Foundation and aims at improving our understanding of the etiology of schizophrenia and bipolar disorder plus their clinical and epidemiological correlations using high dimensional biological investigations and proper analysis. For details on the project see [30-32]. Cases with schizophrenia were identified via the Hospital Discharge Register. Population controls, who had never received a discharge diagnosis of schizophrenia, were selected at random from the national population registers and then group matched to the cases in terms of age, gender and county of residence. All procedures were approved by ethical committees in Sweden and in the US, and all subjects provided written informed consent (or legal guardian consent and subject assent). DNA was extracted from peripheral donated blood at the local medical facilities of the participants.

We obtained, on average, 68.0 million 50 bp reads per sample of which 70.8% could be mapped. After several QC steps we estimated the methylation status of about 27 million autosomal CpGs (all CpGs in the reference genome hg19/ GRCh37). We eliminated 10,483,766 CpGs (mostly located in repeats) showing alignment problems according to an *in silico* alignment experiment plus another 2,735,400 sites showing low read coverage.

MethylPCA performed data reduction in two stages. The first stage consists of combining CpG sites that are very highly correlated (*r* >0.9) because they are largely covered by the same 100–200 bp fragments. In the second stage, we combine the “blocks” from the first stage that are highly correlated (*r* > 0.6) typically due to biological processes.

MethylPCA could combine the remaining 15,558,200 CpGs after QC into 8,822,240 stage (level) 1 blocks, which in turn could be combined into 5,074,538 stage (level) 2 blocks. This represented a 67.3% data reduction. The stage 1 blocks were small (15.6 bp) with high inter-correlations (mean *r* = 0.95) indicating that they involved CpGs in close proximity that were largely covered by the same 100–200 bp fragments. The stage 2 blocks comprised an average of 3.1 CpGs with the largest blocks consisting of >18 CpGs and spanning over 500 bp. This suggested regions seemed to be similarly methylated due to biological processes.

**Figure 3:**
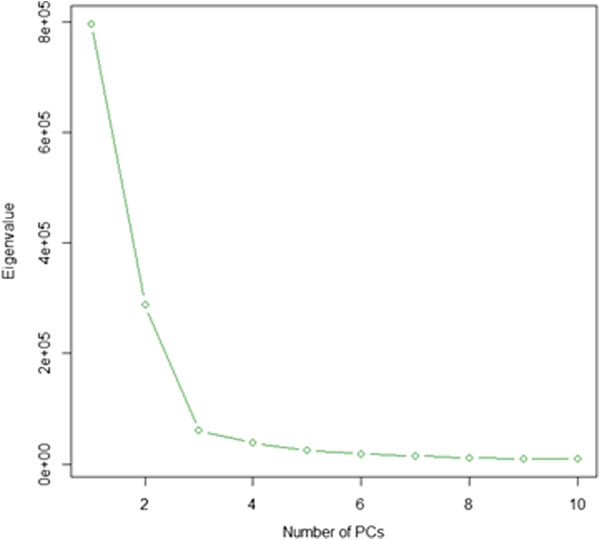
Scree test from the PCA on the on the MBD-seq data set.

**Figure 4:**
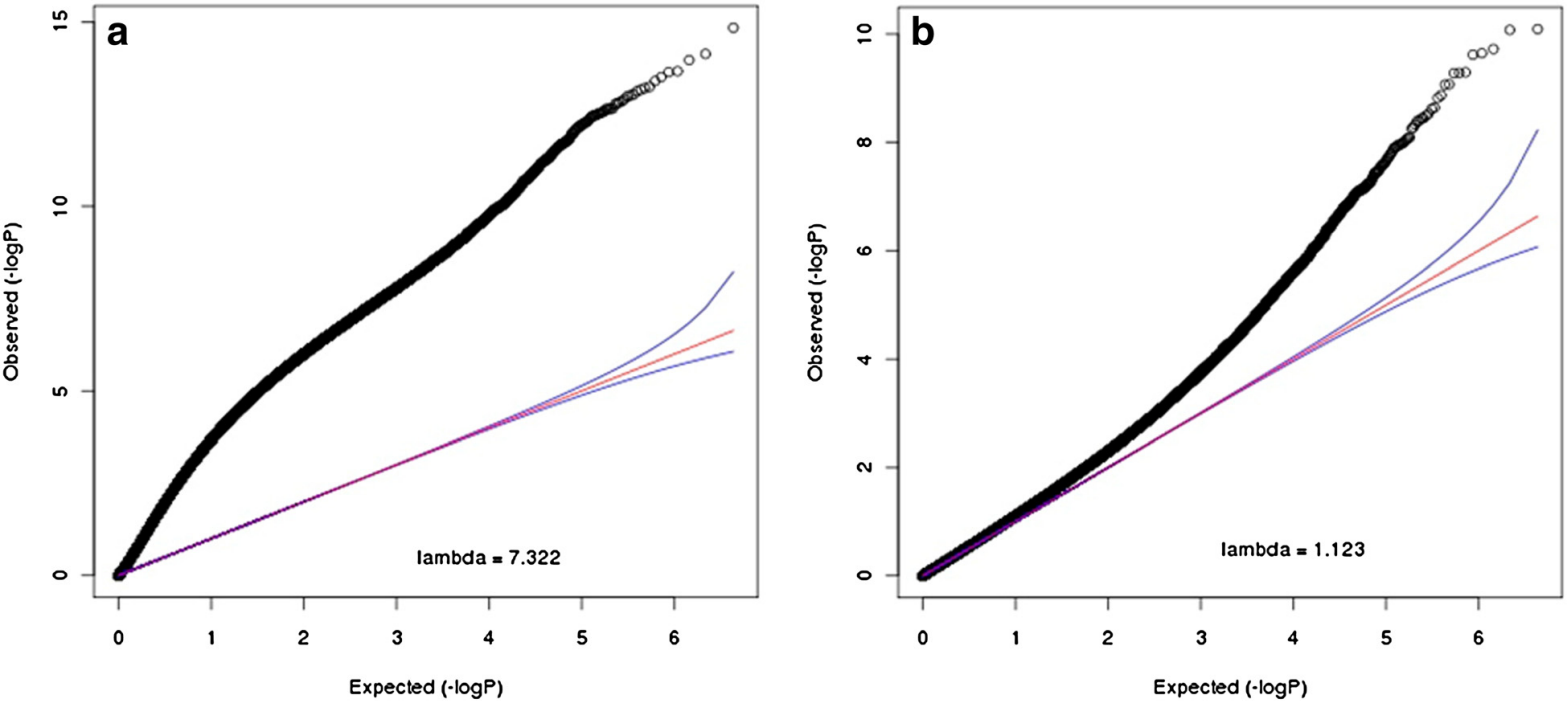
**QQ plot and lambda of the methylome-wide association tests before PCA and after PCA on the schizophrenia data set. **(**a**) shows the QQ plot and lambda without including PCs in the association test. (**b**) shows the QQ plot and lambda including PCs in the association test.

**Figure 5:**
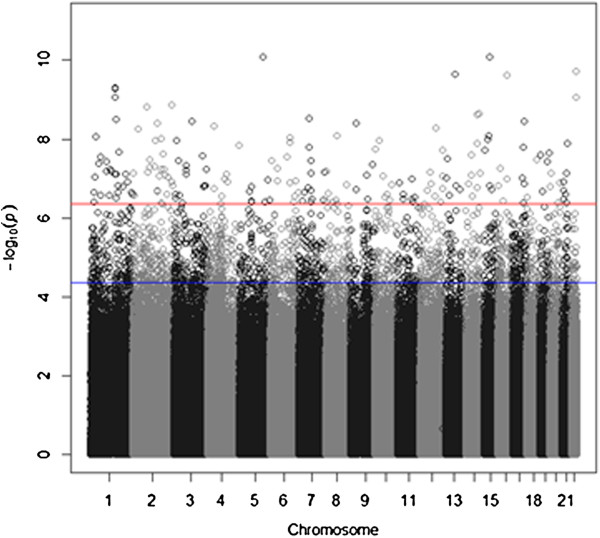
**Manhattan plot for MWAS results on the on the schizophrenia data set.** The red line is *q* value < 0.01 and blue q values < 0.1.

We performed analyses on the 5,074,538 stage 2 blocks. To eliminate possible artifacts related to the lab technical aspects of the data, MethylPCA regressed out 5 technical factors prior to performing PCA: amount of starting material, amount of DNA captured, the duplication status, re-run status and the batch number. Based on a Scree test shown in Figure 3, we choose the top 7 PCs. Age and gender are also included as covariates in the association test. Figure 4 shows the QQ plot and lambda without and with PCs as covariates. The results suggested many confounders (lambda = 7.32) that were subsequently effectively dealt with by regressing out the selected PCs (lambda =1.12). In contrast to the simulated data, the empirical data lambda remained slightly larger than 1. This may be because of disease related cases control differences which need further investigation. The Manhattan plot in Figure 5 indeed suggested a considerable number of effects with 141 blocks being significant when the False Discovery Rate (FDR) is controlled at the 0.01 level, and 25 blocks passing the highly conservative Bonferroni correction (threshold = 1.15 × 10^-8^). The best *p*-values were 10^-11^ to 10^-10^. The top findings were replicated in independent samples using a different technology (targeted bisulfite pyrosequencing) and are discussed in detail elsewhere [Aberg et al. submitted].

The computations were performed on a cluster. Processing the chromosomes in parallel, it took about 14 hours to create the stage 1 blocks and 4 hours to create the stage 2 blocks. We regressed out technical factors prior to the PCA, which took about 2 hours. The PCA was performed by portioning the similarity matrix into chunks of 350 subjects. Using 16 processors of 16 Gb each, the PCA took about 26 hours. The MWAS association test took about 2 hours.

## Discussion

A wide variety of other existing methods can in principle be used to analyze MWAS data. For example, surrogate variable analysis (SVR) developed in the context of microarray experiments [33] can be used to identify and remove unknown latent noise, such as batch effects. However, direct application of these packages [33], may not be practical because of the ultra high dimension of methylation data. Instead, efficient analysis of MWAS data is likely to require tailored computational tools that employs parallel computing, uses a low level programming language for CPU intensive calculations, stores intermediate results to avoid computing the same statistics multiple times or storing results in memory, and uses algorithms specifically designed for high dimensional data.

Our empirical data showed that the risk of false positives in MWAS is very high, likely because of the many differences between cases and controls (e.g. life style, diet, medication use) that affect the methylome. This stressed the need of controlling confounders for which the package MethylPCA was designed. It seems reasonable to assume that if confounders have such pervasive effects on the methylome, the pathogenic processes that cause the disease may also involve many methylation sites. A careful inspection of the PCs (e.g. using the loadings generated by MethylPCA) is important to prevent that disease processes are being regressed out in the MWAS.

As the input methylation data are quantitative values for a set of genomic locations, MethylPCA can be applied to methylation data generated by any assay. Furthermore, because the PCA components can be run independently, in principle it can also be applied to other ultra high dimensional data, such as genome-wide sequence data as long as the specific input format is followed.

## Conclusions

Controlling for confounders in MWAS presents a major computational challenge because of the very large number of possible methylation sites. In this article we introduced MethylPCA that is specifically designed to handle this problem. We tested and demonstrated MethylPCA using simulations and empirical MWAS data from 1,500 subjects. Results showed that MethylPCA effectively controlled for possible confounders.

## Availability and requirements

**Project name:** MethylPCA

**Project home page:**http://www.biomarker.vcu.edu

**Operating systems:** LINUX, MAC OS X, and MICROSOFT WINDOWS

**Programming language:** C++ and R

**Other requirements:** None

**License:** GNU GPL

**Any restrictions to use by nonacademics:** None

## Abbreviations

PCA: Principal component analysis; PC: Principal components; MBD-seq: Methyl-CpG binding domain protein sequencing; MWAS: Methylome-wide association studies.

## Competing interests

The authors declare that they have no competing interests.

## Authors’ contributions

WC designed and co-wrote the software, co-developed the methodology and simulation, carried out the computational experiments, and helped to draft the manuscript. GG co-developed the methodology, discussion of the computational experiments, helped to draft the manuscript. SN, CMH, PKM, PFS, KA collected the samples and processed the methylation data. EJV designed and co-wrote the software, co-developed the methodology and simulation, carried out the computational experiments, helped to draft the manuscript and coordinated the project. All authors read and approved the final manuscript.

## Supplementary Material

Additional file 1 User guide of the software. Click here for file

Additional file 2 **Supplemental material with a brief proof of the Equations (1)-(3), detail on the simulation study, and description of laboratory procedures and QC of the real methylation data.** Click here for file
