# Supplementary material for: MethylPCA: a toolkit to control for confounders in methylome-wide association studies
Source: BMC Bioinformatics. 2013 Mar 2;14:74. doi: 10.1186/1471-2105-14-74 (PMC3599654; doi:10.1186/1471-2105-14-74)
Supplement: Additional file 1 — User guide of the software. [file 1471-2105-14-74-S1.docx]

**MethylPCA**

**User Guide**

**Contents**

1. **Introduction** page 2
2. **Installation and running the software** page 3
3. **Input data format** page 4
4. **Parameter file** page 6

4.1 Mandatory parameters page 6

4.2 Blocks page 7

4.3 PCA page 8

4.4 Association page 10

4.5 Miscellaneous page 10

1. **(Re-)running specific procedures**  page 12
2. **Output files** page 14

6.1 Blocks page 14

6.2 PCA page 14

6.3 Association page 14

6.4 Logs page 16

1. **Examples** page 17

7.1 Run examples and Validate with R page 17

7.2 Simulating example data sets page 18

1. **Introduction**

There are three procedures that MethylPCA can perform:

1) Creating blocks. This procedure combines inter-correlated methylation data from adjacent sites by calculating their mean. Reducing the total number of sites has computational and statistical advantages (e.g., decreased risk of false discoveries, avoid redundancy in the PCA) and the sum of substantially inter-correlated measurements is a more reliable indicator of the underlying signal than the individual measurements separately. Rather than using a sliding window of a pre-determined fixed length, MethylPCA combines sites adaptively based on the observed inter-correlations. Inter-correlations will occur because of biological phenomena. In enrichment based approaches (MBD/MeDIP) inter-correlations can also be the result of neighboring methylation sites being largely covered by the same fragments. To account for these different causes, a two-stage algorithm can be used where first the sites that are largely covered by the same fragments are combined and next the “block” data from the first stage is combined to capture “biological” correlations.

2) PCA. It performs PCA based on input methylation data and output the calculated PC scores, eigenvalues and loadings. The PCA is performed through eigen-decomposition of a much smaller inner product matrix calculated from the methylation data.

3) Association test. It performs association tests with optional supplied covariates. Typical covariates are the PC scores calculated from the PCA procedure. It outputs the test statistics and p-values, as well as a QQ plot.

A parameter file controls which and how procedures are performed. For example, the three procedures can be performed sequentially or individually by removing the parameters corresponding to other procedures in the parameter file. Each procedure has multiple parameters to be set in the parameter file in order to run it properly.

1. **Installation and running the software**

The software assumes that the R software (<http://www.r-project.org/>, R version > 2.13.1) is installed. It does not need additional installation. In case that the compiled executables do not run properly or the users modified the source code, e.g., changing some constants defined in the C++ source files, and need to re-compile, go to the src directory and type:

> bash ./build.sh

This should compile the C++ files using the GNU g++ compiler and put it into the bin directory. Parts of the source files require that the GNU Scientific Library (GSL) installed on the system. Under Windows, users can install MinGW (<http://www.mingw.org/>) and make sure the Windows version of GSL is available. Then users can modify the paths in build_win.bat and run it.

The directory named bin contains all compiled executable programs or R scripts. The directory named examples contains example parameter files and simulated data sets.

To run MethylPCA from the command line window, suppose that the current path in the command line window is the directory examples, type:

> Rscript ../bin/pcaStackJobs.R *parameter_file*

Rscript: Rscript is the command installed with R. If it is not on the path, use the full path of the Rscript command.

pcaStackJobs.R: This is the main R script to read the input parameter file and run all the jobs

*parameter_file*: The file to set parameters, see more detailed descriptions in the section: Parameter file.

To save the console output to a file, type:

> Rscript ../bin/pcaStackJobs.R *parameter_file* > *output_file*

*output _file*: The file to save the console output.

1. **Input data format**

The required input data are the phenotype data and the methylation data. In case the user wants to select sites for inclusion/exclusion from the data, selection files can be provided to indicate which sites will be used.

Phenotype data

The phenotype data are in a comma delimited file with headers and extension .csv, i.e., all variable names on the first row and also separated by commas (called csv file). It specifies the subject IDs, outcome and other optional variables that can be selected to be regressed out from the PCA or association analyses. The order of the columns is in the following fixed order:

Column 1: sampleID (required)

Column 2: outcome (required)

Column 3: covariate 1

Column 4: covariate 2

etc…

The covariates (if provided) can be regressed out prior to performing the PCA and in the association test. The outcome is required for the association test. There is one example file in the example directory: examples/small_data/pheno_file.csv

Methylation data

The methylation data needs to be stored in a directory with subdirectories chr<i>, where <i> is the chromosome number. In each subdirectory chr<i>, every subject has its own data file stored in a csv file with name: <sampleID>.csv. The files have two columns, the first column identifies the site (e.g. CpG coordinate or block number) and the second column contains the methylation measure for that location. Users can see the example methylation data in the directory: examples/small_data/coord.

**NOTE**: Most operating systems have a soft limit for the number of open files. For example, per-user limit defaults to 1024 for UNIX. Because each subject has a separate file for the methylation data, the number of open files is at least the number of the subjects. MethylPCA will exit if the file limit is exceeded. Under Linux, the most effective solution is for the system administrator to change the limit for the nodes where the jobs are running. For Windows 2000/ 2003/XP or Windows 7, the limit is 2048 in our implementation, see <http://msdn.microsoft.com/en-us/library/6e3b887c.aspx>

Selected sites

This is optional. It is a directory with csv format files specifying whether to include/exclude sites when creating larger blocks. There are two columns in the csv file:

Column 1: coord. It specifies the coordinate of the location, e.g., CpG sites, or the blocks number.

Column 2: select. The value 1 means to select the location, 0 means to exclude.

There are example files in the directory: examples/small_data/selected_sites.

1. **Parameter file**

The parameter file controls which and how procedures are performed. Each line sets a parameter with its given value in the following syntax:

<parameter_name> = <parameter value>

The <parameter_name> is a pre-defined label that cannot be changed. The right side is the assigned value specified by the user. There can be spaces before or after the assignment "=". DON'T ADD ANY CHARACTER AT THE END OF EACH LINE, such as ";" or ".", except space, tab character or comments. The parameter file supports comments. Comments begin with "#" and all text after this sign will be ignored. For example, if the # is at the beginning of the line thus the total line will be ignored, see example parameter file: examples/par.txt.

There are several path parameters that need to be specified. MAKE SURE ALL PATH PARAMETERS USE SLASH "/" AS THE PATH SEPARATOR EVEN UNDER WINDOWS AND NO SPACE ON THE PATH. The path can be absolute paths or relative paths which are relative to the current directory of execution.

The next sections describe the parameters for each procedure.

4.1. Mandatory parameters

Mandatory parameters are required for any procedures. They are described in Table 1.

Table 1. Mandatory parameters

| run_in_parallel | Whether or not to run the analyses in parallel: set it to 1 to use the cluster to run jobs in parallel, 0 for serial execution |
| --- | --- |
| chr_begin, chr_end | These two values define the range of chromosomes to be processed. For example, *chr_begin* = 1 and *chr_end* = 22 means using methylation data from chromosome 1 to chromosome 22 |
| methylation_data_directory | The directory of the methylation data |
| phenotype_file | This specifies the name of the phenotype file. |
| RscriptCmd | This specifies the path of the executable command Rscript |
| log_directory | The directory to store log files, intermediate files produced during the execution |

On Windows the jobs will always be executed in serial fashion. Parallel execution assumes Sun Grid Engine or similar batch-queuing systems that use the bash and qsub commands.

Besides the mandatory parameters, each procedure has its own specific parameters described in the following sections.

4.2. Blocks

A maximum of block levels that can be specified per run is 2. More levels of blocks can be generated by running the program iteratively on the created block data from the previous run. The input of the level 1 block-creating procedure is the methylation data specified by *methylation_data_directory*.

The followings are the parameters pertaining to block-creating procedure of level 1

Table 2. Level 1 block parameter

| block1_working_directory | The output directory of the created level 1 block data |
| --- | --- |
| block1_selected_sites_prefix | It specifies the path prefix of the selection files. Therefore the name of the selection files should be <*block1_selected_sites_prefix><chrID>.csv, <chrID>* is the number of the chromosome |
| block1_min_mean_block_cor | This is the threshold for the average inter-block correlation used by the findBlocks.exe program. Recommended values are 0.6 to capture biological correlations and 0.9 to combine sites that are largely covered by the same DNA fragments. |
| block1_min_new_block_cor | This is the threshold for the correlation of a new site vs. sites already in block used by the findBlocks.exe program. Recommended values are 0.3 to capture biological correlations and 0.8 to combine sites that are largely covered by the same fragments. |
| block1_min_n_new_block_cor | This is the threshold used by the findBlocks.exe program. It will stop expanding a block if *block1_min_n_new_block_cor* new sites/blocks have correlation below *block1_min_new_block_cor* with sites already in block. Recommended value is 2 to capture biological correlations or to combine sites that are largely covered by the same fragments. |

To create level 2 block data, it uses the exactly same program but usually run it with different parameter settings. Input data for the second stage blocks are the created output files from the first stage blocks. The meanings of parameters are the same as described in level 1 block parameters, see Table 3.

If the parameter *block1_working_directory* does not exist, no blocks will be created. This is useful for performing PCA without creating blocks. If parameter *block2_working_directory* does not exist but *block1_working_directory* does, then only level 1 block will be created.

Table 3. Level 2 block parameters

| block2_working_directory | the output directory of the created level 2 block data |
| --- | --- |
| block2_selected_sites_prefix | It specifies the path prefix of the selection files. Therefore the name of the selection files should be <*block2_selected_sites_prefix><chrID>.csv, <chrID>* is the number of the chromosome |
| block2_min_mean_block_cor | This is the threshold for the average inter-block correlation used by the findBlocks.exe program. Recommended values are 0.6 to capture biological correlations and 0.9 to combine sites that are largely covered by the same DNA fragments. |
| block2_min_new_block_cor | This is the threshold for correlation of the new site vs. sites already in block used by the findBlocks.exe program. Recommended values are 0.3 to capture biological correlations and 0.8 to combine sites that are largely covered by the same fragments. |
| block2_min_n_new_block_cor | This is the threshold used by the findBlocks.exe program. It will stop expanding block if *block2_min_n_new_block_cor* new sites/blocks have correlation below *block2_min_new_block_cor* with sites already in block. Recommended value is 2 to capture biological correlations or to combine sites that are largely covered by the same fragments. |

4.3. PCA

If blocks are created, the created highest level of block data will be the input methylation data of the PCA procedure. If no blocks are created, it will use the methylation data specified in the parameter *methylation_data_directory*.

The main computing challenge (both in memory and time) of large data sets is the calculation of the inner product matrix XX^T^ (X is centered data matrix and X^T^ is the transpose of X). In this software, we break the calculation of the matrix XX^T^ into chunks and each chunk is calculated separately. These calculations can be executed either in serial or in parallel. Table 4 describes the parameters pertaining to PCA.

The chunk size, i.e., n_sample_chunk, needs to be decided appropriately. Too large chunk size will reduce the number of jobs to be executed in parallel while too small chunk size will increase the I/O because of more repetition of reading of the same sample data. User can first check the memory of the running machine. Then the user can specify an affordable chunk size to load it into memory. For running on clusters, if each node has large enough memory, the parameter n_cores can be set to 1. If the memory of the node can only afford one chunk, use the total number of cores in a node as n_cores to assign one node to the job.

Table 4. PCA parameters

| pca_working_directory | This is the working directory of the PCA procedure. All outputs will be stored in this directory |
| --- | --- |
| regress_out_covariates_before_pca_indices | This specifies the covariates in the *phenotype_file* to be regressed out before performing PCA. It uses a comma separated list of numbers. For example, included_covariates_indices = 1,2,3,4,5,6,7 will include covariate 1 to covariate 7 (columns 3 to 8 because the first two columns are sampleID and outcome). |
| n_sample_chunk | The number of samples (or rows) of each chunk of the XX^T^. As we use double precision for floating point numbers in C++ and on most operating systems this translates to 8 bytes per number, the required giga bytes of memory space per sample is: # of sites × 8 bytes / 10^9^ . Thus, if the maximal number of sites is 6 million, ~166 samples will require about 8Gb of working memory. User need to make sure the *n_sample_chunk* samples can be fitted into the memory. |
| n_cores | This only applied when running on a cluster. The number of cores in a node needed to load the number of sample specified in n_sample_chunk. This only affects the allocation of CPU cores if *run_in_parallel* is set to 1. Set it to the maximal number of cores of a node to avoid memory competition among cores in a single node |
| n_parallel_proc | The number of jobs that can be run in parallel to calculate XX^T^. Multiple chunks can be stacked into one job. These scripts can be submitted to run in parallel if *run_in_parallel* is set to 1 |
| loading_type | This parameter specifies which types of loadings to be calculated: “cov” calculates loadings corresponding to PCA using the covariance matrix, “cor” for loadings using the correlation matrix, “both” for both types of loadings. If *loading_type* is not set, then no loadings will be calculated |
| number_of_PCs_loadings | This specifies the number of top PCs for which the loadings will be calculated |

Even though it is possible to calculate loadings for each principal component, usually we are only interested in the loadings corresponding to the top PC scores. The parameter *number_of_PCs_loadings* allows users to specify the number of top principal components for which loadings will be calculated*.*

If *regress_out_covariates_before_pca_indices* does not exist in the parameter file, PCA will be performed without regressing out any covariates. If it is set, a phenotype file pre_pca_covariates.csv will be generated in the *log_directory*. Then the procedure will regress out the covariates from the methylation data and the regressed data is stored in the directory: <*pca_working_dir>/*residuals. After that, the procedure will perform PCA on the residual data.

If the *pca_working_directory* parameter does not exist, the PCA procedure will not be performed.

4.4 Association

The input of the association tests is the same as the PCA procedure: the input methylation data will be the created highest level of block data if blocks are created, otherwise, it will use the methylation data specified in *methylation_data_directory*. Table 5 shows the parameters for association tests.

If *association_working_directory* does not exist, no association test will be performed.

4.5. Miscellaneous

If several jobs are submitted simultaneously and they all have heavy I/O, the cluster may become very slow. To avoid this I/O jam, a parameter *job_submission_dela*y can delay the submission of jobs for user specified time interval as show in Table 6.

Table 5. Parameters of association tests

| association_working_directory | The output directory of the association tests |
| --- | --- |
| test_label | The test label added to the name of the output. This can be useful if multiple association tests are performed on the same methylation data but with different covariates. Different results can then be distinguished by the test label |
| included_covariates_indices | This specifies the covariates in the phenotype file to be included in the association tests using a comma separated list of numbers. For example, included_covariates_indices = 1,2,3,4,5,6,7,8,9,10 will include covariate 1 to covariate 10 (columns 3 to 12 because the first two columns are sampleID and outcome) |
| included_PCs_indices | This specifies the principal components (PCs) to be included in the association tests. For example, included_covariates_indices = 1,2,3,4,5,6,7,8,9,10 will include PC1 to PC10 |
| included_PCs_types | This specifies which type of PCs to use, “cov” to use PCs based on the covariance matrix, “cor” to use PCs based on the correlation matrix |

Table 6. Miscellaneous Parameters

| job_submission_delay | This parameter specifies the seconds of delay between submitted jobs to the cluster. If not specified, no delay is assumed. This only affects the submission when running under Linux with clusters and *run_in_parallel* is set to 1. |
| --- | --- |

1. **(Re-)running specific procedures**

Rather than running the three procedures (creating blocks, PCA and association test) in a pipeline mode, users may be interested in (re-running) specific procedures. For example, it may be informative to run the association tests with different sets of covariates or principal components or perform the PCA on blocks created with different settings. Generally the user can comment out or remove the sections of parameters so the corresponding procedures will not be executed. Below we illustrate several scenarios of parameter configuration that may help the user to handle different situations.

(Re-)create blocks

Users may want to re-create blocks. For example, one might be interested to re-create stage 2 blocks with different settings but using the same stage 1 blocks. In this case the user only needs to specify the parameter *methylation_data_directory* to the directory of the created level 1 block and set the *block1_working_directory* to the directory storing level 2 blocks (they are level 2 blocks because the input are already level 1 blocks) and change the threshold parameters to the new settings.

Users can also repeat the above process to create any level of blocks higher than level 2, e.g., using level 2 blocks as input and create level 3 blocks. Parameters of other procedures, e.g., the PCA procedure, can be commented out or deleted so only the blocks-creating procedure is executed.

(Re-)run PCA

Users may be interested to perform the PCA with different input data sets of covariates. In this case, the user can set the parameter *methylation_data_directory* to the directory of the created blocks and comment out or remove the block section.

Another situation is that the user wants to (re-)generate the loadings for additional PCs.. In this case, just set the *loading_type* parameter and the *number_of_PCs_loadings*. If the PCs and eigenvalues are already computed successfully, i.e., the files of PCs and eigenvalues exist, they will not be re-calculated. If the user needs to re-calculate the PCs and eigenvalues, make sure the files of calculated PCs and eigenvalues in the directory specified by *pca_working_directory* do not exist.

(Re-)run association test

Users may wish to run additional association tests using the same blocks and PCA. In this case, the user can set the parameter *methylation_data_directory* to the directory of the created blocks and commented out or remove the block section. If the user wants to include PCs as covariates, then KEEP THE PCA SECTION. Since the files of calculated PCs and eigenvalues exist, they will not be re-calculated. However, to avoid re-calculating the loadings, we recommend that the *loading_type* parameter is commented out or deleted. Otherwise the loadings will be re-calculated.

Using batch files from the log directory

An alternative way to rerun (parts of) procedures is to go to the log directory and locate the specific batch files that are executed to perform the task (see the section 6.5 below that explains the log and batch files). Users may read the documentation for individual programs to understand the specific functions of each batch files. This is useful when for some situations where, for example, the descriptives for a specific chromosome were not generated due to an execution error on the cluster. To avoid re-calculating the already existing descriptives for the other chromosomes, the users can go to the log directory to locate the specific batch file for that specific chromosome and rerun it.

1. **Output files**

6.1 Blocks

Block data

The output data are written in the same format as the methylation data. Thus they are stored in a directory with subdirectories chr<i>, where <i> is the chromosome number. In each subdirectory chr<i>, every subject has its own block data files in csv format with the filename: <sampleID>.csv. The files have two columns; the first column gives the block number and the second the methylation measure for that block.

Block information files

Information about the blocks is in the specified block directory in csv files labeled blockInfoChr<i>.csv. The columns are as follows in Table 7:

Table 7. Columns of the block information

| Blocknr | Block number for the relevant chromosome |
| --- | --- |
| *begin* | Begin coordinate (for stage 1 blocks) or stage 1 block number (for stage 2 block) |
| *end* | End coordinate (for stage 1 blocks) or stage 1 block number (for stage 2 block) |
| *all_size* | Total number of sites or blocks in the block |
| *sel_size* | Number of sites or blocks after selection in the block that were combined |
| *meanCor* | The mean correlation in the block. If meanCor = -1 this means that no sites were combined |

6.2. PCA

The PCA outputs are in the directory <*pca_working_directory*> with the following files listed in Table 8.

6.3. Association Tests

Table 9 shows the output from the procedure of the association test.

Table 8. Output files in the PCA procedure

| PCs, eigenvalues | | |
| --- | --- | --- |
| cov_eigenval.csv | Eigenvalues based on the covariance matrix | |
| cov_scores.csv | PC scores based on the covariance matrix | |
| cov_eigenvec.csv | Scaled PC scores (unit variance) based on the covariance matrix. This is also the eigenvectors of XX^T^ | |
| cor_eigenval.csv | Eigenvalues based on the correlation matrix | |
| cor_scores.csv | PC scores based on the correlation matrix | |
| cor_eigenvec.csv | Scaled PC scores (unit variance) based on the correlation matrix. This is also the eigenvectors of XX^T^ with X normalized by the standard deviation. | |
| Loadings on the chromosomes are stored in the following files in the directory <*pca_working_directory*>/loadings/ | | |
| cov_loadings_chr<i>.csv | The loadings on the chromosome based on the covariance matrix, where <i> is the chromosome number | |
| cor_loadings_chr<i>.csv | The loadings on the chromosome based on the correlation matrix, where <i> is the chromosome number | |
| The descriptives of mean and standard deviation of each site/block is stored in <*pca_working_directory*>/descriptives. The assembled PCA input matrices XX^T^ are also stored in the descriptives directory | | |
| des_chr<i>.csv | The first column is the location coordinate, the second and third columns are the mean value and standard deviation across all samples on this location | |
| PCAinput_cov.csv | The matrix XX^T^ for PCA based on covariance matrix | |
| PCAinput_cor.csv | The matrix XX^T^ for PCA based on correlation matrix | |
| sd_PCAinput.csv | The square root of the diagonal elements of the XX^T^. This can be plotted to see the distance of each subject to the origin in the high-dimensional space | |
| The residuals are stored in <*pca_working_directory*>/residuals if there are covariates to be regressed out before PCA | | |
| The chunks of the inner product matrix XX^T^ are stored in <*pca_working_directory*>/descriptives/chunksXXT. | | |
| diag_*<row_begin>*_*<row_end>*.csv | | Diagonal chunks |
| rect_*<row_begin>*_*<row_end>_<col_begin>_<col_end>*.csv | | Off-diagonal chunks |

Table 9. Output files in the association test

| association_<test_label>_ chr<i>.csv | <test_label> is the label set in the parameter file and <i> is the chromosome number. The outputs include the t test statistic and corresponding p-values |
| --- | --- |
| association_<test_label>_qqplot.png | A QQ plot generated with the calculated inflation factor lambda |
| pheno_file_included_covariates_<test_label>.csv | The file contains all covariates to be included in the association test. Users can check this file to make sure the covariates are specified correctly |

6.4 Logs

Log files where MethylPCA writes a record of its activities are stored in the directory *log_directory*. This can be used to diagnose execution errors. The *.bat files are the commands executed to generate all output files.

Table 10. The output log files or intermediate files

| pre_pca_*.bat | These batch files include the pre-pca commands, such as generating blocks, calculate descriptives |
| --- | --- |
| pca_compute_XXT*.bat | batch files calculate the chunks of XXT |
| pca_cov.bat | This file calculates the PCs and eigenvalues based on the covariance matrix |
| pca_cor.bat | This file calculates the PCs and eigenvalues based on the correlation matrix |
| pca_loading_cov*.bat | These batch files calculate the loadings based on the covariance matrix |
| pca_loading_cor*.bat | These batch files calculate the loadings based on the correlation matrix |
| association_prepare_pheno.bat | This file prepares the phenotype file including all covariates for association test |
| association_test*.bat | These batch files do the association tests |
| association_qqplot.bat | This file plots the QQ plot |
| *.o or *.e | For running jobs on a cluster through submission, these are corresponding output files with the suffix .o or .e corresponding to the above batch files |
| sampleIDs.txt | The sample ID file used by individual programs |
| pre_pca_covariates.csv | The file contains the covariates being regressed out before PCA |
| included_covariates_indices.txt | The indices of covariates included in the association test except the PCs. |

For running jobs on clusters, the *.o and *.e files can be used to locate the problems if anything during the execution is wrong. The user can examine these files according to the execution order to locate the first location of error messages.

If some command files (*.bat files) fail to produce the correct results, re-run these command files may solve the problem and save a lot of time comparing to re-running the whole procedure, see the Section: (Re-)running specific procedures.

1. **Examples**

7.1 Run examples and validate with R

In the directory examples, there are example parameter files: par.txt (under Linux) and par_win.txt (under Windows). The only differences between these two parameter files are the way to specify the Rscript command path.

There is also a simulated dataset under the directory examples/small_data.

To test the software on the example data set, users need to make sure the path of the RscriptCmd is correct in the parameter file.

Suppose the current path is the directory: examples. Type:

> Rscript ../bin/pcaStackJobs.R par.txt

or

> Rscript ../bin/pcaStackJobs.R par_win.txt

MethylPCA can be validated using R functions prcomp() for PCA and lm() for association tests. The *validation* parameter can be used to perform the validation processes. Set it to 1 to validate with pure R functions, and 0 without validation. If the parameter dose not exists, no validation is performed.

Note that this validation should only be applied on a small data set, otherwise using pure R functions will either cause memory problem or the computing time is huge for PCA, which is exactly the reason why we develop this software.

The validation procedure compares pure R vs. C++/R implemented in this package. When the validation is performed, validation results can be found either in <pca_workdir>/validation_pca.txt or in <association_workdir>/validation_association.txt

The output of the PCA validation in validation_pca.txt should be something like this:

[1] "norm of differences in eigenvalues: cov"

[1] 7.97549e-10

[1] "norm of differences in PC scores: cov"

[1] 7.93737e-09

[1] "norm of differences in loading: cov"

[1] 2.942016e-12

[1] "norm of differences in eigenvalues: cor"

[1] 5.43983e-13

[1] "norm of differences in PC scores: cor"

[1] 1.780305e-10

[1] "norm of differences in loading: cor"

[1] 2.251253e-12

This verifies that the calculation of the C++/R program has the same results as those from the R function prcomp() up to precision errors.

The output of the validation for association tests in validation_association.txt should be something like this:

[1] "norm of differences in test statistics"

[1] 1.736119e-12

[1] "norm of differences in pvalues"

[1] 1.049352e-12

This verifies that the calculation of the association tests using C++ is the same as using R function lm().

7.2 Simulating example data sets

The example data set is generated by the R script simulateMethyData.R in the bin directory. If interested, users can play with the parameters in the R code to generate other simulated data. In the example directory, it can be run as follows:

> Rscript ../bin/simulateMethyData.R <methylation_data_dir> <phenotype_file> <selected_sites_prefix>

Rscript need to be replaced with its full path if it is not in the system path. Here is the actual command used to generate the example data set in this package assuming the current directory is: examples:

> Rscript ../bin/simulateMethyData.R "./small_data/coord/" "./small_data/pheno_file.csv" "./small_data/selected_sites/sel_chr"
