# Supplementary material for: MethylPCA: a toolkit to control for confounders in methylome-wide association studies
Source: BMC Bioinformatics. 2013 Mar 2;14:74. doi: 10.1186/1471-2105-14-74 (PMC3599654; doi:10.1186/1471-2105-14-74)
Supplement: Additional file 2 — Supplemental material with a brief proof of the Equations (1)-(3), detail on the simulation study, and description of laboratory procedures and QC of the real methylation data. [file 1471-2105-14-74-S2.docx]

Supplemental material for the paper:

**MethylPCA:**

**A toolkit to control for confounders in methylome-wide association studies**

Content

1. Principal component analysis (PCA) when *n* << *p* page 2
2. Simulation study page 4
3. MBD-seq MWAS in 1,500 samples page 6

**1. Principal component analysis (PCA) when *n* << *p***
Suppose X is the *n* × *p* methylation data matrix for *n* subjects at *p* sites. We assume that X has been centered by subtracting the mean of all observations of the corresponding site from the original observation. The sample covariance matrix $C=\frac{1}{n-1}X^{T}X$ is a *p* × *p* matrix. Suppose *λ_1_*, *λ_2_*, …, *λ_r_* are the positive eigenvalues in descending order and *v_1_*, *v_2_*, … *v_r_*  are the corresponding orthonormal eigenvectors of C, with subscript *r* being the rank of X. Then *PC_i_* = X*v_i_* is the *n*-dimensional column vector of the *i*th principle component (PC) scores of methylation data across *p* sites for the *n* subjects, where the elements in *v_i_* are also called the loadings of *PC_i_*. When *n* << *p* it can be computational infeasible to conduct the PCA through eigen-decomposition of matrix C. Instead, we can calculate *PC_i_* by using the *n* × *n* matrix $M=\frac{1}{n-1}XX^{T}.$ Suppose *α_1_*, *α_2_*, …, *α_r_* are the positive eigenvalues in descending order and *u_1_*, *u_2_*, …*u_r_* are the corresponding orthonormal eigenvectors of M. Let U = [*u_1_*, *u_2_*, …*u_r_*] and V = [*v_1_*, *v_2_*, … *v_r_*]. If U and V are properly chosen when they are not unique, we have (see[1])

*λ_i_* = *α_i_* (1)

and ${PC}_{i}=X v_{i} =u_{i}\sqrt{{\left( N-1 \right)\alpha}_{i}}$, *i =* 1*,*$\cdots$*, r*. (2)

So the PC scores can be calculated using *u_i_* and *α_i_*. Similarly we have

$v_{i}=\frac{1}{\sqrt{\left( N-1 \right)\alpha_{i}}} X^{T}u_{i}=\frac{1}{\left( N-1 \right)\alpha_{i}} X^{T}{PC}_{i}$, *i =* 1*,*$\cdots$*, r,* (3)

Therefore, the loadings *v_i_* can also be calculated from *u_i_* and *α_i_*. EIGENSOFT [2] employs similar method to calculate principle components.

We describe a brief proof of equations (1)-(3). For convenience, we ignore the constant 1/(n-1) in matrix $C=\frac{1}{n-1}X^{T}X$ and work on the matrix $X^{T}X$, because the same conclusion can be easily extended to $\frac{1}{n-1}X^{T}X$. We still use *λ_i_* and $v_{i}$ to denote the positive eigenvalue and the corresponding unit-length eigenvector of $X^{T}X$, *α_i_* and $u_{i}$ to denote the positive eigenvalue and the corresponding unit length eigenvector of $XX^{T}$. From the singular value decomposition of X, we have X = ADB^T^, where A = [*a*_1_*, a*_2_*, …, a_n_*], B = [*b_1_, b_2_, …, b_p_*], *a_i_* is a *n*-dimensional column vector (*i* =1,2,…, *n*), *b_i_* is a *p*-dimensional column vector (*j* =1,2,…, *p*), and A is *n × n* and B is *p × p* and both are orthogonal matrices. D is a *n × p* diagonal matrix with only top *r* diagonal elements *d_1_, d_2_,* …*, d_r_* positive. We have X^T^X = BD^T^A^T^ADB^T^ = BD^T^DB^T^ = BD^T^DB^-1^. Here D^T^D is a *p × p* diagonal matrix with positive values $d_{1}^{2}, d_{2}^{2}, {\ldots, d}_{r}^{2}$ on the diagonal. Since matrix B is orthogonal, we have (X^T^X) *b_i_* =$d_{i}^{2}b_{i}$(*i* = 1, 2, …, *r* ). This means that $d_{i}^{2}$ and *b_i_*, are the *i*-th positive eigenvalues and corresponding eigenvectors of X^T^X, respectively. Similarly we can have XX^T^ = ADD^T^A^-1^, and (XX^T^) *a_i_* =$d_{i}^{2}a_{i}$ This means that $d_{i}^{2}$ and *a_i_*, are the *i*-th positive eigenvalues and corresponding eigenvectors of XX^T^. Because D^T^D and DD^T^ have the same *r* positive eigenvalues, we can have *λ_i_* = *α_i_* = $d_{i}^{2}$ and equation (1) is approved. Now we choose *u_i_* = *a_i_*, *v_i_* = *b_i_*, *i* = 1, 2, …, *r*. To prove equation (2) we have X*v_i_* = X*b_i_* = ADB^T^*b_i_* = *a_i_d_i_* = *u_i_d_i_* = $u_{i}\sqrt{\alpha_{i}}$. *i*= 1, 2, …, *r*. To prove equation (3), we have X^T^*u_i_* = X^T^*a_i_* = BD^T^A^T^*a_i_* = *b_i_d_i_* = *v_i_d_i_* = $v_{i}\sqrt{\alpha_{i}}$. *i*= 1, 2, …, *r*.

**2. Simulation study**

For simplicity, the subscript *i* representing the *i*th subject is omitted in the following equations.

**I. The correlation between the case-control status and the *k*th confounding factor of continuous type**

$\text{corr}(F_{k},y)=\frac{\mathrm{cov}(F_{k},y)}{\sqrt{Var(F_{k})}\sqrt{Var(y)}}=\frac{\mathrm{cov}(b_{k}y+m_{k},y)}{\sqrt{Var(b_{k}y+m_{k})}\sqrt{Var(y)}}$.

Because *m_k_* and *y* are independent, we have

$$\begin{matrix} \text{corr}(F_{k},y)=\frac{b_{k}\mathrm{cov}(y,y)+ \mathrm{cov}(m_{k},y)}{\sqrt{b_{k}^{2}Var(y)+Var(m_{k})}\sqrt{Var(y)}}=\frac{b_{k}\mathrm{cov}(y,y)}{\sqrt{b_{k}^{2}Var(y)+Var(m_{k})}\sqrt{Var(y)}} \\ =\frac{b_{k}Var(y)}{\sqrt{b_{k}^{2}Var(y)+Var(m_{k})}\sqrt{Var(y)}}=\frac{1}{\sqrt{1+\frac{Var(m_{k})}{b_{k}^{2}Var(y)}}} \end{matrix}$$

If we assume the same number of cases and controls, then *Var*(*y*) = ½ × (1 – ½) = ¼. So we have $\text{corr}(F_{k},y)=\frac{1}{\sqrt{1+\frac{4\times Var(m_{k})}{b_{k}^{2}}}}=\frac{1}{\sqrt{1+\frac{4\times\sigma^{2}}{b_{k}^{2}}}}$

**II. The correlation between the case-control status and the *k*th confounding factor of dichotomous type**

From the conditional probability and assuming the same number of cases and controls, we have

Pr (*F_k_* = 1, *y* = 1) = Pr (*F_k_* = 1| *y* = 1) × Pr(*y* = 1) = *p_1_*/2,

Pr (*F_k_* = 0, *y* = 1) = Pr (*F_k_* = 0| *y* = 1) × Pr(*y* = 1) = (1 – *p_1_*)/2,

Pr (*F_k_* = 1, *y* = 0) = Pr (*F_k_* = 1| *y* = 0) × Pr(*y* = 1) = *p_2_*/2,

Pr (*F_k_* = 0, *y* = 0) = Pr (*F_k_* = 0| *y* = 0) × Pr(*y* = 1) = (1 – *p_2_*)/2,

So Pr(*F_k_* = 1) = (*p_1_ + p_2_*)/2, Pr(*F_k_* = 0) = 1 – (*p_1_ + p_2_*)/2, therefore

E(*F_k_*) = (*p_1_ + p_2_*)/2,

Var(*F_k_*) = E( (*F_k_* – E(*F_k_*))^2^) = (*p_1_ + p_2_*)/2 × (1 – (*p_1_ + p_2_*)/2),

Pr (*F_k_ y* = 1) = *p_1_*/2, Pr (*F_k_ y* = 0) = 1 – *p_1_*/2, E(*F_k_ y*) = *p_1_*/2,

cov(*F_k_*, *y*) = E(*F_k_ y*) - E(*F_k_*) E(*y*) = (*p_1_ – p_2_*)/4,

$$\begin{matrix} \text{corr}(F_{k},y)=\frac{\mathrm{cov}(F_{k},y)}{\sqrt{Var(F_{k})}\sqrt{Var(y)}}=\frac{(p_{1}-p_{2})/4}{\sqrt{(p1+p2)/2\times(1-(p1+p2)/2)}\times\frac{1}{2}} \\ =\frac{p_{1}-p_{2}}{\sqrt{2\times(p1+p2)-(p1+p2)^{2}}} \end{matrix}$$

**3. MBD-seq MWAS in 1,500 samples**

Our study includes 750 schizophrenia cases and 750 controls, as well as 75 technical duplicates. This study is part of a large ongoing project entitled “A Large-Scale Schizophrenia Association Study in Sweden”. The project is supported by grants from NIMH and the Stanley Foundation and aims at improving our understanding of the etiology of schizophrenia and bipolar disorder plus their clinical and epidemiological correlates using high dimensional biological investigations and proper analysis. For details on the project see [3-5]. Cases with schizophrenia were identified via the Hospital Discharge Register. Population controls, who had never received a discharge diagnosis of schizophrenia, were selected at random from the national population registers and then group matched to the cases in terms of age, gender and county of residence. All procedures were approved by ethical committees in Sweden and in the US, and all subjects provided written informed consent (or legal guardian consent and subject assent). DNA was extracted from peripheral donated blood at the local medical facilities of the participants.

### Laboratory procedures

We used the MethylMiner kit from Invitrogen (Carlsbad, CA), that employs MBD2 protein-based enrichment of the methylated DNA fraction, followed by single end sequencing (50 bp read length) on the Applied Biosystems SOLiD next generation sequencing platform (Life Technologies, Foster City, CA). Methods were standard and based upon manufacturer's recommendations. This approach which combines an MBD enrichment protocol with next generation sequencing is typically referred to as MBD-seq.

### QC and data processing

*Alignment:* The sequenced reads were aligned to the human genome (build hg19/GRCh37) using BioScope (Life Technologies), a multithread application that aligns in color-space and takes full advantage of the increased ability of SOLiD two-base encoding to identify sequencing errors [6]. We used a seed-and-extend approach combined with local alignment and multiple schemas. The seed was 25 bases. Rather than considering the entire extension, local alignment may improve sensitivity by finding the maximum similarity score between the observed sequence and a substring of the reference sequence. A maximum of 2 color space mismatches were allowed in the seed (e.g. as 2 color call matches are required to change the base call, a SNP will have two color call mismatches). If the seed could not be mapped, we tried a second schema by moving the seed from base 1 to base 15.

*Coverage estimation:* To obtain a methylation measurement we calculated coverage for the 28,217,444 million CpG sites in the reference genome (hg19/ GRCh37) plus a further 2,385,935 million CpG sites that could be created by known SNPs (dbSNP build 132). A standard procedure is to count the number of sequence reads covering the CpG which, because the methylation of any CpG in the entire fragment could lead to its capture by MBD protein binding, where the read length is sometimes extended to account for the fact that the fragments will be longer than the reads. However, because not all fragments will have exactly the same specified size and the fragment pool obtained after shearing may not be identical to the pool that gets successfully sequenced (e.g. smaller fragments may be more likely to get pulled down by the enrichment protocol), this procedure can be imprecise. Furthermore, particularly in large scale studies there may be (stochastic) variation in the fragment size distribution across samples. Thus, rather than assuming an identical pre-determined fragment size for all fragments and samples, we estimated the fragment size distribution for each sample empirically from the actual sequencing data.

A limitation of commonly used methods for estimating fragment size distributions, e.g. those used in ChIP-seq peak finding algorithms [7-8], is that they make strong parametric assumptions about this distribution and do not take advantage of the specific features of MBD-seq data where we know exactly where methylation can occur. We therefore developed a non-parametric method that uses isolated CpGs to estimate the fragment size distribution empirically from the sequencing data. To validate our method we sequenced paired-end libraries in eight mice [9]. Using the successfully aligned read pairs, we obtained the “observed” fragment size distribution by subtracting the start positions of the two reads. Next, we excluded one read from each pair and used our estimator. Results showed very close correspondence (correlation was 0.996) between the estimated and the observed distribution obtained from paired-end reads, suggesting that our method worked well.

The estimated fragment size distributions are used to calculate the probability for each read that the fragment it is tagging covers the CpG under consideration. Coverage for each CpG is calculated as the sum of the probabilities that all fragments in its neighborhood cover the CpG. For example, this probability would equal 1.0 for fragments with reads starting within 50bp of the CpG, but would be ≤1.0 for fragments with reads starting further away. Coverage is affected by the total number of used reads per sample that is a function of the lab protocol (e.g. degree of multiplexing) rather than methylation. Our estimates were therefore standardized using the total number of reads that remained after QC. Coverage estimates can be further normalized by the CpG density [10]. This is typically done to obtain a better estimate of the actual amount of methylation [11]. However, in association analyses we essentially compare the mean in cases and controls. As this mean difference remains unchanged by corrections based on CpG density (i.e. the same constant will be added for cases and controls), for sake of simplicity we did not use such a normalization step.

*Eliminating sites with alignment problems:* CpG sites in loci that are problematic in terms of alignment need to be eliminated prior to analysis as coverage estimates will be confounded with alignment errors. For example, repetitive elements constitute about 45% of the human genome. Reads may be difficult to align to these loci because of their high sequence similarity. To identify problematic sites we conducted an *in silico* experiment. We first generated all 2.86 billion possible 50 bp single-end reads for the human reference genome (build hg19/GRCh37), with each 50bp sequence starting 1bp downstream of the previous sequence. For example, the sequence of *in silico* read 1 was identical to the sequence of position 1 to 50 on chromosome 1 of the reference, read 2 was identical to positions 2 to 51, etc. In the perfect scenario, aligning these reads to the reference genome should show that each CpG is covered by 50 reads. CpG sites with coverage < 40 or >60 were eliminated from further analyses.

*Data reduction by combining correlated sites:* MethylPCA performed data reduction in two stages. The first stage consists of combining CpG sites that are very highly correlated (*r* >0.9) because they are largely covered by the same 100-200 bp fragments. In the second stage, we combine the “blocks” from the first stage that are highly correlated (*r* > 0.6) typically due to biological processes.

MethylPCA could combine the 15,558,200 CpGs remaining after QC into 8,822,240 stage 1 blocks, which in turn could be combined into 5,074,538 stage 2 blocks. This represented a 67.3% data reduction. The stage 1 blocks were small (15.6 bp) with high inter-correlations (mean *r*=0.95) indicating that they involved CpGs in close proximity that are largely covered by the same 100-200 bp fragments. The stage 2 blocks comprised an average of 3.1 CpGs with the largest blocks consisting of >18 CpGs and spanning over 500 bp. This suggested regions seemed to be similarly methylated due to biological processes.

### Association testing

We performed association analyses on the 5,074,538 stage 2 blocks. To eliminate possible artifacts related to the lab technical aspects of the data, MethylPCA regressed out 6 variables prior to performing the PCA.

**References**

1. Gower JC: **Some Distance Properties of Latent Root and Vector Methods Used in Multivariate Analysis**. *Biometrika* 1966, **53**:325-338.

2. Price AL, Patterson NJ, Plenge RM, Weinblatt ME, Shadick NA, Reich D: **Principal components analysis corrects for stratification in genome-wide association studies**. *Nat Genet* 2006, **38**(8):904-909.

3. Bergen SE, O'Dushlaine CT, Ripke S, Lee PH, Ruderfer D, Akterin S, Moran JL, Chambert KD, Handsaker RE, Backlund L *et al*: **Genome-wide association study in a Swedish population yields support for greater CNV and MHC involvement in schizophrenia compared to bipolar disorder**. *Molecular Psychiatry* In press.

4. Schizophrenia Psychiatric Genome-Wide Association Study Consortium: **Genome-wide association study of schizophrenia identifies five novel loci**. *Nature Genetics* 2011, **43**:969-976.

5. International Schizophrenia Consortium: **Common polygenic variation contributes to risk of schizophrenia and bipolar disorder**. *Nature* 2009, **460**:748-752.

6. McKernan KJ, Peckham HE, Costa GL, McLaughlin SF, Fu Y, Tsung EF, Clouser CR, Duncan C, Ichikawa JK, Lee CC *et al*: **Sequence and structural variation in a human genome uncovered by short-read, massively parallel ligation sequencing using two-base encoding**. *Genome Res* 2009, **19**(9):1527-1541.

7. Pepke S, Wold B, Mortazavi A: **Computation for ChIP-seq and RNA-seq studies**. *Nat Methods* 2009, **6**(11 Suppl):S22-32.

8. Zhang Y, Liu T, Meyer CA, Eeckhoute J, Johnson DS, Bernstein BE, Nusbaum C, Myers RM, Brown M, Li W *et al*: **Model-based analysis of ChIP-Seq (MACS)**. *Genome Biol* 2008, **9**(9):R137.

9. van den Oord E, Bukszar J, Rudolf G, Nerella S, McClay J, Consortium SS, Xie K, Åberg K: **Estimation of CpG coverage in whole methylome next-generation sequencing studies**. *Submitted* 2012.

10. Harris RA, Wang T, Coarfa C, Nagarajan RP, Hong C, Downey SL, Johnson BE, Fouse SD, Delaney A, Zhao Y *et al*: **Comparison of sequencing-based methods to profile DNA methylation and identification of monoallelic epigenetic modifications**. *Nat Biotechnol* 2010, **28**(10):1097-1105.

11. Down TA, Rakyan VK, Turner DJ, Flicek P, Li H, Kulesha E, Graf S, Johnson N, Herrero J, Tomazou EM *et al*: **A Bayesian deconvolution strategy for immunoprecipitation-based DNA methylome analysis**. *Nat Biotechnol* 2008, **26**(7):779-785.
